# Association between serum carcinoembryonic antigen and cardiometabolic risks: Implication for cardiometabolic prevention

**DOI:** 10.3389/fendo.2023.1113178

**Published:** 2023-02-23

**Authors:** Chia-Hao Chang, Hsu-Huei Weng, Yu-Chih Lin, Chia-Ni Lin, Tung-Jung Huang, Mei-Yen Chen

**Affiliations:** ^1^ Department of Nursing, Chang Gung University of Science and Technology, Chiayi, Taiwan; ^2^ Department of Diagnostic Radiology, Chang Gung Memorial Hospital, Chiayi, Taiwan; ^3^ Department of Family Medicine, Chang Gung Memorial Hospital, Yunlin, Taiwan; ^4^ Department of Laboratory Medicine, Chang-Gung Memorial Hospital, Linkou, Taiwan; ^5^ Department of Medical Biotechnology and Laboratory Science, Chang Gung University, Taoyuan, Taiwan; ^6^ Department of Pulmonary and Critical Care, Chang Gung Memorial Hospital, Yunlin, Taiwan; ^7^ School of Nursing, Chang Gung University, Taoyuan, Taiwan; ^8^ Research Fellow, Department of Cardiology, Chang Gung Memorial Hospital, Chiayi, Taiwan

**Keywords:** tumor marker, carcinoembryonic antigen (CEA), oxidative stress, 1hydroxypyrene (1-OHP), malondialdehyde (MDA), cardiometabolic diseases (CMDs), unhealthy lifestyle

## Abstract

**Background:**

Serum carcinoembryonic antigen (CEA) is a biomarker commonly used to detect colorectal cancer. CEA levels are affected by many factors, including cardiometabolic diseases, such as cardiovascular diseases (CVDs) and diabetes. Cardiometabolic diseases and cancer share a similar pathological inflammatory pathway, which correlates with an unhealthy lifestyle. Hence, establishing an adequate CEA cut-off value might be a valuable reference for developing precision healthcare programs for cardiometabolic disease prevention. This study aimed to investigate the association between cardiometabolic risks and serum CEA and the underlying factors.

**Methods:**

A community-based, cross-sectional study was conducted between March and December 2021 on the western coast of Taiwan. Lifestyle data were assessed using a structured questionnaire. The cardiometabolic biomarkers, serum CEA, urine malondialdehyde, and 1-hydroxypyrene were quantified by the central laboratory of the collaborating hospital. Chi-square and binary multivariable logistic regression implemented in R version 4.0.2 were used to identify factors defining the risk of high serum CEA levels.

**Results:**

A total of 6,295 adult residents without cancer-related diseases completed the study. The mean age was 48.6 (SD = 16.4) years, 56% were female, 32% had metabolic syndrome, and 23% and 10% had CVDs and diabetes, respectively. Multivariate logistic regression showed that age ≥ 65 years, male sex, alcohol consumption, smoking, infrequent use of dental floss, fewer remaining teeth, CVDs, diabetes, and oxidative stress were significantly associated with serum CEA ≥ 3 ng/mL. The discriminatory performance of the area under the receiver operating characteristic curve was 0.75 (0.73–0.76), showing that this model was suitable for distinguishing high CEA levels.

**Conclusion:**

Our findings highlight the importance of understanding cardiometabolic diseases, unhealthy lifestyles, and oxidative stress, which contribute to high serum CEA. This study demonstrates that CEA, a well-known tumor marker, can help the early detection and prevention of cardiometabolic diseases *via* personalized lifestyle modification.

## 1 Introduction

Recently, researchers studying the interplay between carcinogenesis and cardiometabolic diseases (CMDs) have focused on reactive oxygen species (ROS) and redox imbalance (1–3). ROS, such as oxygen, nitrogen, and sulfur, are highly reactive derivatives of oxygen metabolism and are considered normal cellular metabolites. ROS can be used as a biomarker for oxidative stress. ROS plays a double-edged role in cellular damage and protection (4, 5). Levels of oxidative stress biomarkers, such as urinary 1-hydroxypyrene (1-OHP) and malondialdehyde (MDA), were significantly higher in patients with colorectal cancer (CRC) and correlated with aging, smoking, liver diseases, and CMDs (2, 6). ROS activate the pro-inflammatory signaling pathway and cytokines that induce endothelial cell dysfunction and cause vascular smooth muscle migration and hyperplasia. Serial reactions result in atheroma formation and further CMDs such as hypertension, heart disease, stroke, and type 2 diabetes (2, 7, 8). Serum carcinoembryonic antigen (CEA) is an inflammatory biomarker commonly used to detect colorectal cancer. CEA levels are affected by many factors, including CMDs.

CMDs are recognized as systemic diseases induced by dysregulation of systemic inflammation, immunity, and metabolism and have been shown to have direct effects on atherosclerotic plaques, insulin resistance, and diabetes (9, 10). Furthermore, CMDs and cancer are the leading causes of morbidity and mortality worldwide (11, 12) and in Taiwan (13, 14), with similar biological mechanisms related to the inflammation process, as well as many modifiable risk factors, such as smoking, low vegetable, and fruit intake, obesity, physical inactivity, hypertension, dyslipidemia, and non-modifiable aging, as well as genetic factors (9, 15, 16). According to the literature, most CMDs and cancers can be prevented through modifiable risk factors, such as reduced tobacco and alcohol consumption, changes in an unhealthy diet, and physical activity (9, 11, 15). Additionally, cardiometabolic risks can be detected early, before progression to CMDs, *via* primary health examination. Cardiometabolic risks are a cluster of risk factors such as abdominal obesity, impaired glucose tolerance, elevated blood pressure, triglycerides, and low high-density lipoprotein cholesterol, which increases the risk of CMDs. Furthermore, the presence of three or more of these risk factors is known as metabolic syndrome (MetS) (9, 14, 17).

Except for fecal occult blood tests, serum carcinoembryonic antigen (CEA) is commonly used for the early detection of CRC in many annual health examination settings. CEA, a surface glycoprotein mainly found in epithelial and mucus-secreting cells of the colon, participates in cancer invasion and metastasis (18, 19). CEA is a malignant transformation and chronic inflammation marker and was first identified as a colon cancer antigen; it was previously used as a prognostic marker in CRC and monitoring response to therapy (20, 21). Previous studies showed increased serum CEA levels in CRC and chronic diseases, such as hyperglycemia, CVDs, and type 2 diabetes (17, 19, 22). European cardiologists recently reported that CEA was associated with the severity of heart failure outcomes, including cardiovascular morbidity and mortality (3, 18, 23). The underlying mechanism might be due to imbalanced oxidative damage and endoplasmic reticulum stress production, triggering redox imbalance and increasing oxidative damage to proteins, lipids, and DNA (24)

However, no cut-off reference value is available to distinguish high serum CEA levels in clinical practice. Traditionally, clinicians used serum CEA for tumor detection in CRC and to monitor the response to further treatment. Few studies have linked CEA levels and cardiometabolic risks in primary prevention to mitigate CMDs pathogenesis and progression. Hence, we aimed to investigate the possible modifiable factors associated with high serum CEA levels and establish a cut-off value of serum CEA levels for the prevention of CMDs among adults in rural communities.

## 2 Materials and methods

### 2.1 Design and population

This study was part of a series of health promotion programs designed to explore health needs and provide tailored health care for adults in rural areas. Community-based annual health screening was conducted in collaboration with a local hospital between March and December 2021 in western coastal Yunlin County, Taiwan. Participants were selected using convenience sampling. The inclusion criteria were as follows: (1) age ≥ 20 years, (2) the ability to complete questionnaires in a Mandarin or Taiwanese dialect *via* a face-to-face interview, and (3) agreement to participate in the study after providing informed consent. The exclusion criteria were as follows: (1) inability to complete the questionnaires, (2) inability to perform self-care or walk independently, (3) diagnosis of cancer-related diseases, and (4) incomplete health surveys or laboratory data.

### 2.2 Procedure and ethical considerations

This study conformed to the principles outlined in the Declaration of Helsinki and was approved by the Institutional Review Board of the Research Ethics Committee (IRB no: 202000109B0C101). All participants were informed about the study’s purpose, procedures, benefits, and potential risks agreed to participate, and signed an informed consent form. Five registered nurses were recruited as research assistants and trained by the investigators. The one-on-one questionnaire interview included health-related lifestyle behaviors and was established from a previous study (25). The questionnaire designed was based on the relationships between a healthy lifestyle and anti-inflammatory reactions, such as adequate diets, regular exercise, and oral hygiene are benefits for cardiometabolic health (15, 25). Blood and urine samples were drawn and stored according to the standard procedure by the central laboratory of the collaborating hospital.

### 2.3 Measurements

#### 2.3.1 Demographic and health history

Demographic and health history included age, sex, level of education (years of education received), and self-reported comorbidities diagnosed by a physician (diabetes, hypertension, heart disease, and stroke).

#### 2.3.2 Substance use was assessed

Substance use was assessed: (a) regular alcohol consumption at least three times per week and (b) cigarette smoking, with responses categorized as “never” vs. “yes: former or current user.”

#### 2.3.3 Healthy diet

Healthy diet was assessed using the frequency of at least three portions of vegetables and two portions of fruit per day, with responses categorized as “less: never or seldom” and “often: usually or always.”

#### 2.3.4 Regular exercise

Regular exercise was based on whether the participants usually or always (often) exercised for > 30 min, at least three times per week, or seldom or never (less) engaged in exercise.

#### 2.3.5 Oral health

Oral health was measured as follows: (a) the number of natural teeth and fixed dentures were self-reported, and (b) frequency of using dental floss before bed with responses of “less: never or seldom” or “often: usually or always.”

#### 2.3.6 Cardiometabolic risk factors

Cardiometabolic risk factors were based on the national standard (14), including the presence of five physiological biomarkers: (a) elevated central obesity (waist circumference) in males and females > 90 and 80 cm, respectively, (b) elevated systolic/diastolic blood pressure > 130/85 mmHg, (c) low serum high-density lipoprotein-cholesterol (HDL-C) in males and females < 40 and 50 mg/dL, respectively, (d) elevated serum fasting blood glucose (FBG) > 100 mg/dL, and (e) elevated serum triglyceride (TG) levels > 150 mg/dL. MetS were defined by the presence of three or more risk factors.

#### 2.3.7 Carcinoembryonic antigen

Carcinoembryonic antigen (CEA, ng/mL) was measured by electrochemiluminescence immunoassay (ECLIA) on Roche Cobas e801 analyzer. Instead of using reference intervals published in manufacturers’ package inserts, we used CEA ≥ 3.0 ng/mL as the cut-off value for the high serum level group based on previous studies, considering age, sex, and smoking habits (18, 22, 26).

#### 2.3.8 Urine 1-hydroxypyrene and malondialdehyde

Urine 1-hydroxypyrene (1-OHP) and malondialdehyde (MDA) (μg/g CRE): Spot urine samples were collected and sent to the central laboratory of the collaborating hospital for analysis. Urinary 1-OHP was analyzed using ultra-performance liquid chromatography-tandem mass spectrometry (UPLC-MS/MS) and urinary MDA was quantified using standard thiobarbituric acid reactive substances (TBARS) assay. The urinary creatinine concentration was used for urinary 1-OHP and MDA adjustments (5, 6).

### 2.4 Statistical analysis

This study used the R version 4.0.2 software (The R Foundation for Statistical Computing, Vienna, Austria) for data analysis, including (1) Chi-square and t-tests performed to confirm the differences according to the CEA category (CEA < 3 or CEA ≥ 3 ng/mL); (2) binary multivariable logistic regression used to identify the factors affecting the risk of CEA ≥ 3; (3) to measured effects of data discrepancies. The dataset was randomly divided into two subsets using the Caret R package, with 80% of the data (n = 5036) in the training subset and the remaining 20% (n = 1259) in the validation set. In the training cohort, significant variables (p < 0.05) were selected for binary multivariable logistic regression analysis in the univariate analysis. The model of the training cohort used backward elimination processes to predict the risk of CEA levels ≥ 3 ng/mL. The fitted model was applied to the training and validation subsets. The probability of CEA levels ≥ 3 was calculated based on the beta coefficients of the training subset. The area under the receiver operating characteristic (ROC) curve (AUC) values of the training and validation datasets were calculated using the pROC R package, and (4) To evaluate overfitting, the logistic regression model was fitted to the 1000 bootstrap samples, and the corresponding values for the AUC were calculated. The results were averaged to provide a final bootstrap estimate for AUC optimism. The differences in the values for the averaged AUC and training subset AUC provided an estimate of optimism.

## 4 Results

### 4.1 Demographic characteristics

A total of 6,295 participants aged ≥ 20 years who completed the community annual health examination were included, of whom 3507 (56%) were female and 1204 (19.1%) were classified as having a CEA ≥ 3 ng/mL (**Table 1**). The mean age of the participants was 48.6 years (SD = 16.4, range 20–90 years), with more than three-quarters of those aged < 65 years.

**Table 1:** Univariate analysis of factors associated with higher serum level of CEA (N=6295).

Variables		Total	CEA^12^ <3	CEA≥3	χ^2^/t	p-value
		n (%)	n (%) / M±SD	n (%) / M±SD		
Age (years)	<65	4952 (79)	4197 (82)	755 (63)	225.9	<0.001
	≥65	1343 (21)	894 (18)	449 (37)		
Gender	Female	3507 (56)	3033 (60)	474 (39)	161.1	<0.001
	Male	2788 (44)	2058 (40)	730 (61)		
Healthy diet	Often^1^	3361 (53)	2765 (54)	596 (50)	9.1	<0.01
	Less	2934 (47)	2326 (46)	608 (50)		
Alcohol	Never	5277 (84)	4398 (86)	879 (73)	128.6	<0.001
	Yes^2^	1018 (16)	693 (14)	325 (27)		
Smoking	Never	5462 (87)	4597 (90)	865 (72)	288.8	<0.001
	Yes	833 (13)	494 (10)	339 (28)		
Exercise	Often^1^	3414 (54)	2775 (55)	639 (53)	0.8	0.37
	Less	2881 (46)	2316 (45)	565 (47)		
Dental floss	Often^1^	3203 (51)	2746 (54)	457 (38)	99.5	<0.001
	Less	3092 (49)	2345 (46)	747 (62)		
Remaining teeth	≥20	5143 (82)	4300 (84)	843 (70)	135.9	<0.001
	<20	1152 (18)	791 (16)	361 (30)		
WC (cm)^3^	< 80/90	3735 (59)	3060 (60)	675 (56)	6.6	0.01
	≥ 80/90	2560 (41)	2031 (40)	529 (44)		
BP (mmHg)^4^	< 130/85	3505(56)	2966(58)	539(45)	71.8	<0.001
	≥ 130/85	2790(44)	2125(42)	665(55)		
FBG (mg/dL)^5^	< 100	3886 (62)	3312 (65)	574 (48)	124.5	<0.001
	≥ 100	2409 (38)	1779 (35)	630 (52)		
HDL-C (mg/dL)^6^	> 40/50	5111 (81)	4164 (82)	947 (79)	6.3	0.01
	≤ 40/50	1184 (19)	927 (18)	257 (21)		
TG (mg/dL)^7^	< 150	5083 (81)	4187 (82)	896 (74)	38.4	<0.001
	≥ 150	1212 (19)	904 (18)	308 (26)		
MetS^8^	< 3 risk factors	4309 (68)	3611 (71)	698 (58)	75.7	<0.001
	≥ 3 risk factors	1986 (32)	1480 (29)	506 (42)		
CVD^9^	No	4838 (77)	4084 (80)	754 (63)	169.5	<0.001
	Yes	1457 (23)	1007 (20)	450 (37)		
Diabetes	No	5677 (90)	4709 (92)	968 (80)	161.0	<0.001
	Yes	618 (10)	382 (8)	236 (20)		
MDA (μg/g CRE)^10^		0.47 (0.71)	0.45 (0.70)	0.57 (0.72)	4.9	<0.001
1-OHP (μg/g CRE)^11^		0.09 (0.17)	0.08(0.16)	0.12 (0.18)	7.2	<0.001

^1^ Often: usually/always; Less: never/seldom;

^2^ Yes: former or current user.

^3^ Waist circumferences: male/female;

^4^ Blood pressure: systolic/diastolic;

^5^ Fasting blood glucose;

^6^ High-density lipoprotein-cholesterol: male/female;

^7^ Triglyceride;

^8^Metabolic syndrome;

^9^ cardiovascular diseases: hypertension or heart disease;

^10^ malondialdehyde;

^11^ 1-hydroxypyrene;

^12^ carcinoembryonic antigens.

### 4.2 Factors associated with high serum CEA level

Univariate analysis showed that male sex (p < 0.001), age ≥ 65 years (p < 0.001), alcohol consumption (p < 0.001), cigarette smoking (p < 0.001), less consumption of vegetables and fruits (p < 0.01), reduced use of dental floss (p < 0.001), and fewer than 20 natural teeth (p < 0.001), were significantly associated with high serum CEA levels (**Table 1**). To compare participants with or without cardiometabolic risks, those with increased abdominal obesity (p < 0.01), elevated systolic/diastolic blood pressure (p < 0.001), elevated serum FBG (p < 0.001), low HDL-C (p < 0.01), elevated TG (p < 0.001), and MetS (p < 0.001) were significantly associated with higher serum CEA level. Participants who reported having been diagnosed with CVDs (hypertension or heart disease, p < 0.001) and diabetes (p < 0.001) by a physician were classified as having high serum CEA levels. Owing to the lack of reference values for urine 1-OHP and MDA concentration levels, we further compared the mean differences and found that higher levels of urine MDA (p < 0.001) and 1-OHP (p < 0.001) were significantly associated with high serum CEA (**Table 1**).

**Figure 1:**
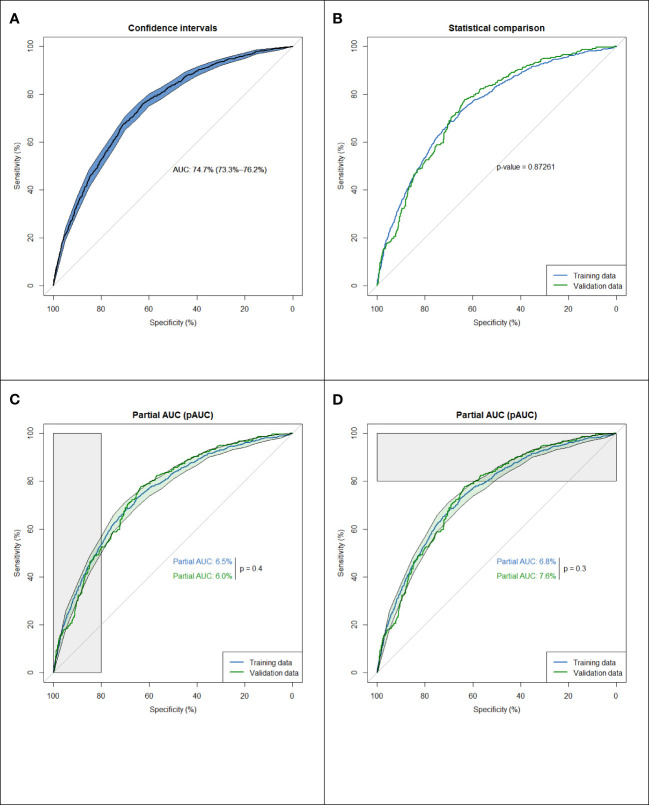
Assessing the discrimination of a fitted logistic model, via the ROC curve. **(A)** Discriminatory performance of the full model (n=6295). **(B)** Comparison of ROC curves for the training data (n=5036) and the validation data (n=1259). **(C)** Average sensitivity, between 80%-100% specificity values. A little disparity (p=0.4) in the model's performance in prospective testing under high true negative rate. **(D)** Average specificity, between 80%-100% sensitivity values a little disparity (p=0.3) in the model's performance in prospective testing under ospective high true positive rate.

The multivariable logistic regression model shows that the estimated odds of participants with ages ≥ 65 [odds ratio (OR) = 2.25, 95% confidence interval (CI) 1.88–2.70], male sex (OR = 1.71, 95% CI 1.47–1.98), alcohol consumption (OR = 1.25, 95% CI 1.04–1.5), cigarette smoking (OR = 3.11, 95% CI 2.56–3.77), less using dental floss (OR = 1.32, 95% CI 1.14–1.53), fewer remaining teeth (OR = 1.32, 95% CI 1.11–1.57), CVDs (OR = 1.41, 95% CI 1.20–1.67), diabetes (OR = 1.81, 95% CI 1.48–2.21), urine MDA (OR = 1.14, 95% CI 1.04–1.24), and 1-OHP (OR = 1.90, 95% CI 1.33–2.73) were significantly associated with higher serum CEA levels (**Table 2**). Overall, the discriminatory performance of the full model revealed an AUC of 0.747 (0.733–0.762) (**Figure 1A**), indicating the suitability of this model in identifying participants with high serum CEA levels.

**Table 2:** Logistic regression analysis of factors associated higher serum level of CEA^6^.

Variables		Full Model (All=6295)		Training Model(n=5036)	
		OR (95% CI)	p-value	OR (95% CI)	p-value
Age (years)	<65	Ref	<0.001	Ref	<0.001
	≥65	2.25 (1.88-2.70)		2.34 (1.91-2.86)	
Gender	Female	Ref	<0.001	Ref	<0.001
	Male	1.71 (1.47-1.98)		1.67 (1.42-1.99)	
Healthy diet	Often ^1^	Ref	0.08	Ref	<0.01
	Less	1.16 (0.98-1.36)		1.28 (1.07-1.53)	
Alcohol	Never	Ref	0.02	Ref	0.03
	Yes^2^	1.25 (1.04-1.5)		1.25 (1.02-1.54)	
Smoking	Never	Ref	<0.001	Ref	<0.001
	Yes	3.11 (2.56-3.77)		3.14 (2.54-3.90)	
Exercise	Often ^1^	Ref	0.89	–	–
	Less	1.01 (0.88-1.16)		–	
Dental floss	Often ^1^	Ref	<0.001	Ref	<0.001
	Less	1.32 (1.14-1.53)		1.35 (1.15-1.59)	
Remaining teeth	≥20	Ref	<0.01	Ref	0.03
	<20	1.32 (1.11-1.57)		1.24 (1.02-1.50)	
CVD^3^	No	Ref	<0.001	Ref	<0.001
	Yes	1.41 (1.20-1.67)		1.47 (1.23-1.78)	
Diabetes	No	Ref	<0.001	Ref	<0.001
	Yes	1.81 (1.48-2.21)		1.76 (1.40-2.20)	
MDA (μg/g CRE)^4^		1.14 (1.04-1.24)	<0.01	1.10 (1-1.21)	0.05
1-OHP (μg/g CRE)^5^		1.90 (1.33-2.73)	<0.001	1.87 (1.26-2.76)	<0.01

^1^ Often: usually/always; Less: never/seldom; ^2^ Yes: former or current user.

^3^ cardiovascular diseases; hypertension or heart disease; ^4^ malondialdehyde; ^5^1-hydroxypyrene; ^6^ carcinoembryonic antigens.

### 4.3 Training and validation

From the dataset of the 6295 participants, we used 80% of the entries as training data (n = 5036) and 20% for testing (n = 1259). To clarify the potential confounding variables in the training set, backward elimination by binary multivariable logistic regression was used to assess the association between CEA ≥ 3 and various factors. A comparison of the ROC curves for the training and validation data indicated an area difference of 0.003 (0.749–0.746, p = 0.87), reflecting a small disparity between the two curves and suggesting a small decay in the model performance in prospective testing (**Figure 1B**).

The partial area under the ROC curve (pAUC) allows us to focus on the area of interest on the left/right side of the ROC plot (**Figures 1C**, **D**), i.e., average sensitivity, between 80–100% specificity values, and average specificity, between 80–100% sensitivity values. **Figure 1C** shows a slight disparity (p = 0.4) in the model’s performance in prospective testing under a high valid negative rate. However, **Figure 1D** shows a slight disparity (p = 0.3) in the model performance in prospective testing under a high true positive rate. To validate this difference, bootstrap processes were repeated 1000 times, and the results were averaged to provide an optimum correction for an AUC of 0.003 (AUC range = 0.758–0.79), indicating the lack of overfitting.

## 5 Discussion

To the best of our knowledge, this study is the first to investigate the relationship between serum CEA ≥ 3 (ng/mL), oxidative stress biomarkers, unhealthy lifestyle factors (such as poor oral hygiene, smoking, and fewer remaining teeth), and cardiometabolic risks. The present study provides valuable findings for further interventional studies and evidence-based lifestyle modifications for the early detection and prevention of cardiometabolic risks. Three crucial findings were obtained from this study. First, a high prevalence of cardiometabolic risks was observed, which was significantly associated with high serum CEA levels. Second, an unhealthy lifestyle was significantly associated with a high serum CEA level. Third, the oxidant stress biomarkers 1-OHP and MDA were also positively associated with high serum CEA levels.

### 5.1 Serum CEA can be used for the early detection of cardiometabolic risks

The present study demonstrated that a high prevalence of cardiometabolic risk is significantly associated with high serum CEA levels. For instance, 44%, 41%, 38%, 32%, 23%, and 10% of participants had elevated blood pressure, central obesity, elevated FBG level, MetS, CVDs, and diabetes, respectively. In addition, almost all cardiometabolic risk factors were significantly associated with high serum CEA levels. Similar to previous studies, CEA levels did not only increase in CRC but were also higher in some chronic diseases, especially CVDs, MetS, and diabetes (17, 19, 22). This finding implies that clinicians can use serum CEA as a useful biomarker for the early detection of cardiometabolic risks and unhealthy lifestyles rather than solely as a tumor marker for CRC. Furthermore, if participants had smoked and suffered from CRC and cardiometabolic risks, it is important to clarify which factor primarily contributed to their high serum CEA levels. Huang et al. (19) demonstrated that postoperative serum CEA levels could not predict survival in CRC patients with type 2 diabetes. Type 2 diabetes and cigarette smoking influence serum CEA levels, which may cause a prognostic bias.

The commonly used clinical threshold value for tumor detection is serum CEA ≥ 5 (ng/mL) (19, 26, 27). However, based on previous studies [18,22] and considering smoking, age, and sex, we used CEA ≥ 3 (ng/mL) as a cut-off value and used this model to distinguish high serum CEA levels based on the area under the ROC curve (AUC). Considering that nearly one-third of the total death rate is caused by CMDs, the increase was higher than ever of cancer in Taiwan (13, 14). The findings presented herein could guide further studies for the early detection and prevention of cardiometabolic risks using serum CEA levels as a useful biomarker.

### 5.2 Serum CEA levels can be reduced by adopting a healthier lifestyle

Despite male sex and aging factors, the present study revealed that an unhealthy diet (e.g., inadequate amounts of vegetables and fruits), alcohol consumption, and smoking significantly increased serum CEA levels. Furthermore, the present study indicated that urinary 1-OHP and MDA levels correlated with higher serum CEA levels. A possible mechanism might be due to ROS and redox imbalance. Evidence supports that ROS activates the inflammation process and induces endothelial cell dysfunction, causing vascular smooth muscle migration and hyperplasia (10, 15, 16). These findings agreed with previous studies showing that aging, substance use, and an unhealthy diet correlated with elevated CEA levels (24, 28, 29). On the other hand, some dietary compounds and metabolites, such as components of the Mediterranean diet pattern (rich in whole grains, fish, fruits, and vegetables), directly affect HDL-C composition and enhance anti-inflammatory and vasoprotective properties (9, 15, 30).

Increased oxidative stress plays a significant role in cardiometabolic risk as well as the initiation and progression of atherosclerosis. However, adopting a healthy diet and engaging in regular exercise are associated with the prevention of CMDs by reducing the inflammatory process (10, 31, 32). Therefore, the American Heart Association (AHA) guidelines suggest that all adults consume a healthy diet that emphasizes the intake of vegetables and fruits, in addition to exercising for at least 150 min per week (9). However, the present study did not accurately account for the effect of regular exercise on serum CEA levels, which could have been insufficiently characterized, as our questionnaire only asked whether the participants exercised for > 30 min, at least three times per week. This criterion does not meet the AHA recommendation of 150 mins per week. Further studies should use more precise tools to gauge exercise behavior.

Moreover, the present study showed that infrequent use of dental floss before bed and tooth loss with < 20 remaining teeth were associated with high serum CEA levels. Several studies have shown that lifestyle modifications, including oral hygiene, regular exercise, a healthy diet, and weight control, are important in managing cardiometabolic risks (9, 33). This finding echoed those of previous studies reporting that poor oral hygiene facilitates infections by *Helicobacter pylori* and other bacteria that increase the inflammatory reaction *via* dental plaque, which in turn increases the possibility of periodontal disease, type 2 diabetes, and CVDs (33–35). Hence, it is worth initiating further interventional studies for adults with high serum CEA through personalized healthcare, including smoking and alcohol cessation, maintaining an adequate number of natural teeth through good oral hygiene, and following a Mediterranean diet.

### 4.3 Strengths and limitations

This is the first study involving large-scale reporting of the relationship between the traditional use of serum CEA as a tumor marker and cardiometabolic diseases, identifying determinant factors associated with higher serum CEA levels. Moreover, we used R version statistical analysis to identify CEA levels ≥ 3 as a reasonable cut-off value to distinguish factors associated with high serum CEA, which can be applied to clinical and community settings for early detection of unhealthy lifestyles and providing personalized health promotion programs. However, this study had some limitations. First, it was conducted in only one county, which may limit the generalizability of the findings. Second, the health-related behavior questions were mostly self-reported, which might generate measurement bias and affect the study findings. For instance, the frequencies relative to vegetable consumption and exercise might be inaccurate. In addition, owing to the coronavirus disease pandemic, the number of remaining teeth was self-reported and not counted by the research assistants. Furthermore, our study lacks deep probing into the history of cardiometabolic diseases, such as prescribed medications for hypertension, heart disease, and diabetes. Hence, the prevalence of cardiometabolic risks may be underestimated.

## 6 Conclusion

A high prevalence of cardiometabolic risk factors was associated with high serum CEA levels. Furthermore, unhealthy lifestyles and oxidative stress biomarkers contributed to high serum CEA levels. CEA ≥ 3 ng/mL was a meaningful threshold value for classifying significant risk factors. Therefore, in addition to being a tumor marker for CRC, CEA could be used in clinical and community settings for the early detection and prevention of CMDs through individualized lifestyle modifications.

## Data availability statement

The original contributions presented in the study are included in the article/supplementary material. Further inquiries can be directed to the corresponding author.

## Ethics statement

The studies involving human participants were reviewed and approved by the institutional review board of the Chang Gung Memorial Hospital Foundation (IRB no: 202000109B0C101). The patients/participants provided their written informed consent to participate in this study.

## Author contributions

C-HC and H-HW contributed to statistical analysis. M-YC and C-HC conceived and designed the study and interpreted the data. C-NL, H-HW, Y-CL, and T-JH collected the data and contributed to the study direction. All authors contributed to the article and approved the submitted version.
